# Prophylactic Hydroxyurea Treatment Is Associated with Improved Cerebral Hemodynamics as a Surrogate Marker of Stroke Risk in Sickle Cell Disease: A Retrospective Comparative Analysis

**DOI:** 10.3390/jcm11123491

**Published:** 2022-06-17

**Authors:** Brian R. Peine, Michael U. Callaghan, Joseph H. Callaghan, Alexander K. Glaros

**Affiliations:** 1 Clinical Research Institute, College of Medicine, Central Michigan University, 1280 S. East Campus Dr., Mount Pleasant, MI 48859, USA; calla1mu@cmich.edu (M.U.C.); aglaros@dmc.org (A.K.G.); 2 Children’s Hospital of Michigan, 3901 Beaubien Blvd, Detroit, MI 48201, USA; 3 Accounting Program, School of Business Administration, Oakland University, 201 Meadow Brook Road, Rochester, MI 48309, USA; callaghan1954@gmail.com

**Keywords:** sickle, ischemic, stroke, trans cranial doppler, average maximum mean velocity, Hydroxyurea

## Abstract

Sickle cell disease (SCD) increases the incidence of childhood stroke eighty-fold. Stroke risk can be estimated by measurement of the blood velocity through the middle cerebral artery (MCA) using transcranial doppler ultrasound (TCD). A high MCA blood velocity indicates increased stroke risk due to cerebral vasculopathy, and first-line treatment to prevent primary or recurrent strokes in high-risk children with SCD has classically been chronic blood transfusions. Research has more recently shown that many of these patients may safely transition from transfusions to oral hydroxyurea (HU) treatment while maintaining a decreased risk of stroke. However, the effect on stroke risk of truly prophylactic HU treatment beginning in infancy, prior to the onset of cerebral vasculopathy, is less well understood. Our retrospective study aimed to document the long-term effects of HU treatment compared with no HU treatment in children with SCD, using TCD measurements as our primary outcome and a surrogate marker of stroke risk. Our results showed that when accounting for age-related variability and duration of treatment, prophylactic HU treatment was independently associated with lower TCD MCA velocities compared with no HU treatment, providing further evidence supporting its early initiation for patients with SCD.

## 1. Introduction

Sickle cell disease (SCD) affects approximately 100,000 Americans and is most prevalent in Black populations, occurring in about 1 out of every 365 African American births [1]. SCD is a multisystem disease that can damage virtually any organ in the body, including the brain. It is one of the most common causes of stroke in children (ages 2–16) throughout the world. Children with SCD have a stroke incidence of 240 per 100,000 per year, compared with 3 per 100,000 strokes per year in the general childhood population [2]. Among children with SCD, it is vital to identify those who have the highest risk of stroke and to provide effective prophylactic treatment.

In a large observational cohort study published in 1997, Adams and colleagues found that MCA velocity, as measured using transcranial doppler ultrasound (TCD), was an adequate predictor of stroke risk in SCD children. Of the 315 participants in the study, only two had been reported to receive any transfusions, which would have impacted the results. The remainder were reported to have received no treatment during this study. They demonstrated that among patients with a time averaged mean of maximum velocities (TAMX) of <1.7 m/s (normal), 98% remained stroke-free after 40 months. For those with a TAMX between 1.7 and 2 m/s (conditional), this rate dropped to 93%, and with a TAMX of >2 m/s (abnormal) it dropped to about 60%. These TAMX values corresponded with a 1%, 3.5%, and 10% annual stroke risk, respectively, with repeat exams providing a significantly increased predictive power [3,4]. Annual TCDs for children with SCD have since become the standard of care and have dramatically reduced stroke incidence when coupled with appropriate primary prophylaxis.

Primary prophylaxis has, over time, taken on two forms: lifelong chronic blood transfusions to maintain a reduced percentage of sickle hemoglobin (HbS) or hydroxyurea (HU) therapy initiated after an initial period of chronic blood transfusions. The former evolved from much older evidence supporting transfusions to prevent recurrent stroke, and was supported by the STOP trial, which was terminated early due to a 92% reduction in stroke risk among those patients with abnormal (TAMX > 2 m/s) TCDs who were treated with chronic blood transfusions [5,6,7,8]. Subsequent investigation in the pediatric population has shown appropriate TCD screening and the initiation of transfusion prophylaxis to reduce overall stroke risk prior to age 18 from 11% to about 2%, with a significant return of stroke risk following transfusion program cessation [9,10,11]. Despite the proven efficacy of indefinite chronic transfusion therapy in reducing acute ischemic stroke (AIS) risk, it carries significant complications, risks, and costs, namely associated with the long-term morbidity caused by transfusion-related hemosiderosis. Identifying safe discontinuation strategies has thus long been a research priority, with the only identified alternative being HU, but only in certain settings.

HU was first approved for treatment in SCD patients after it was discovered to increase the levels of fetal hemoglobin (HbF) and improve the downstream clinical outcomes and hematologic parameters [12]. Multiple observational studies have demonstrated that HU prescribed to treat clinical severity also decreases TCD velocities [13,14,15,16,17]. In the landmark TWiTCH trial (TCD with Transfusions Changing to Hydroxyurea), in which children with SCD and previously abnormal TCD velocities were transitioned to HU treatment after at least 12 months of chronic blood transfusions, the investigators concluded that HU is non-inferior to chronic transfusions for maintaining low TCD velocities and managing stroke risk. The investigators concluded that it was safe to transition patients to HU therapy if they first received blood transfusions for at least 12 months and showed no evidence of severe intracerebral vasculopathy [18]. Preliminary evidence from an ongoing trial in Nigeria indicates that HU therapy may be as effective as chronic transfusions for decreasing stroke risk, even in children with abnormal TCD velocities who have not previously received chronic transfusion therapy, with a stroke incidence among HU patients of 0.76 per 100 person-years, significantly lower than the 10.7 per 100 person-years of the standard group and comparable to the 0.9 per 100 person-years of the transfusion group in the STOP trial [7,16]. Although some statistical power is lost when comparing results with those of a 20-year-old study, the results are promising.

A recently published randomized clinical trial compared the efficacy of moderate-dose (20 mg/kg/day) and low-dose (10 mg/kg/day) HU in primary stroke prevention among children with SCD [19]. Conducted in Nigeria, where regular blood transfusions cannot be feasibly done, this study demonstrated that stroke risk between moderate-dose and low-dose HU treatment was not significantly different. However, the moderate-dose HU group had a lower incidence of all-cause hospitalizations. They concluded that their findings provided support for the use of low-dose HU among SCD children with increased stroke risk.

HU has previously been shown to decrease TCD velocities and act as an adequate alternative to safely transition off chronic transfusions in SCD children with controlled intracerebral vasculopathy. However, long-term trends following the use of prophylactic HU, particularly the incidence of first abnormal TCD relative to those children with SCD who refuse or do not tolerate HU, remain less well understood. In this retrospective chart review, we sought to answer the question of HU’s effectiveness as a primary prophylaxis for stroke prevention in children with SCD. We evaluated the long-term effect of prophylactic HU on TCD velocities relative to velocities in children receiving chronic transfusion (CT) therapy or no treatment. We tested the primary hypothesis that—while controlling for age as a covariate using mixed methods modelling—maximum tolerated dose hydroxyurea therapy in children with SCD would be associated with a decrease of >10% MCA TAMX velocity (surrogate for stroke risk) compared with children receiving no treatment.

## 2. Materials and Methods

### 2.1. Study Type: We Conducted a Retrospective Cohort Chart Review

Selection of patients: We included all patients previously diagnosed with hemoglobin SS disease (HbSS) seen at the Comprehensive Sickle Cell Clinic at the Children’s Hospital of Michigan (CHM) who had at least one TCD. The ages at the time of data extraction (14 July 2020) ranged from 2 to 25 years, although all TCDs were performed when those patients were between the ages 2 and 16.

**Figure 1:**
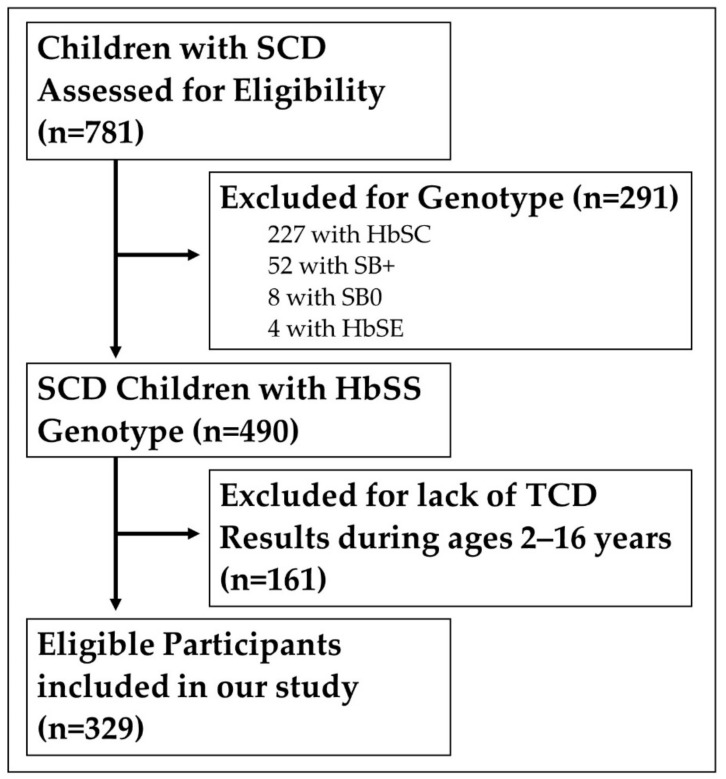
Patient inclusion flow chart. Outline of reasons for patient inclusion or exclusion in our study. SCD genotypes other than HbSS were excluded because they carry varying stroke risks and there were too few with TCD results to perform an analysis with the genotype as a covariate. Patients were required to have results from a minimum of one TCD for inclusion.

Inclusion and exclusion criteria: All patients with HbSS seen at the SCD center at the CHM who had at least one TCD on record were included in the study. Out of 781 patients in the records, 452 were excluded for either having a different sickle cell disease genotype or not having TCD data. There was an insufficient number of children of other SCD genotypes who had the TCD results necessary to perform an analysis with the genotype as a covariate. Of the few children with alternate genotypes who had TCD results, none were receiving HU treatment, leading to the decision to only include HbSS patients. Lack of TCD data was most often due to patient non-compliance with recommended annual TCD screenings [20]. After exclusions, 329 patients were included in our study (Figure 1).

TCD methodology: Measurements at the center were performed using a GE Logic E9 ultrasound machine, including a vector array transducer with a color doppler and duplex with no angle correction. TAMX was calculated using the values of the trace averaged over the time interval shown on the spectrum, as follows: TAMX = sum(Vt) from t1 to t2/(t2 − t1).

Measurement of study factors: The primary study factor was the use of HU at the time of TCD. The maximum tolerated dose (mg/kg bw per day) was recorded for each patient. Adherence to HU was assessed by recording the ranges of the mean corpuscular volume (MCV) and fetal hemoglobin (HbF) during HU use.

Measurement of outcome factors: The outcome factors were the right and left MCA TAMX velocities (m/s) recorded using TCD. The right and left MCA TAMX velocities were analyzed together. Each value was analyzed by whether or not HU was being taken at the time of the TCD, and each value was assigned a duration of time (years) between the start date of HU and the date of TCD. TCDs were obtained for screening at the baseline visit for each patient.

Ethics requirements: This retrospective chart review required no more than a minimal risk to the patients whose data we were collecting. It was reviewed and approved by the Detroit Medical Center (DMC) IRB, contingent on approval or exemption by the Central Michigan University (CMU) IRB. It was subsequently reviewed and deemed exempt by the CMU IRB, fulfilling the DMC IRB requirement.

### 2.2. Data Analysis

The data were extracted to Microsoft Excel 2016 (Microsoft, Redmond, WC, USA). *p*-values for all of the analyses were considered significant below an alpha level of 0.05. Extracted data included age, gender, HU use (Y/N), HU start date, HU end date, maximum tolerated HU dose (mg/kg bw), range of HbF during HU use, range of MCV during HU use, history of chronic transfusions (Y/N), start date and end date for chronic transfusions, date of TCD, right MCA TAMX velocity, and left MCA TAMX velocity (Table 1). MCA TAMX velocities were also categorized according to normal (<1.7 m/s), conditional (between 1.7 and 2.0 m/s), and abnormal (>2.0 m/s) parameters established in previous studies, and a chi-squared analysis was conducted. TAMX velocities were initially divided into two groups. Group 1 included data obtained from patients receiving HU at the time of TCD. Group 2 included data obtained from patients not receiving HU at the time of TCD. For further analysis, TCD measurements were further divided into HU, CT, or no treatment. We compared the groups using MCA TAMX blood flow velocity as the measured outcome. We first compared TAMX velocities measured during HU treatment with those measured in the absence of HU treatment (regardless of other interventions), using a *t*-test to determine statistical significance. Second, we compared velocities measured during HU treatment, CT treatment, and no treatment by conducting an ANOVA test and constructing a box and whisker plot. Subsequently, we factored in HU adherence by measuring MCA TAMX velocities as a function of HbF, conducting an ANOVA test and constructing a box and whisker plot.

Following this initial analysis, a secondary analysis was performed to account for age as a covariate. The 329 unique patients in our study were distributed by no treatment, CT, and HU, with a maximum of ten episodes per patient. The CT group was compiled of patients from the original HU and no HU groups, including patients who had at least one TCD while receiving CT therapy either before or after treatment with HU. Each patient’s MCA TAMX was measured twice at each TCD, right (R) and left (L) sides, doubling the number of measures. We assumed that each side measured by TCD represented hemispherical stroke risk, not a repeated measure of general stroke risk. The number of TCDs varied by patient and was set to a maximum of ten episodes to assure that each treatment level was represented at each episode. The repeated time measures within a single subject were assumed to be correlated.

Several reasonable structures were first estimated and the best fitting one was selected before the main model effects were derived. The Toeplitz model, which estimates a constant covariance (correlation) over a given lag but no pattern over different lags, was found to be the best fitting. Toeplitz is better over the four fit statistic measures, while the other variance–covariance models perform relatively poorly, including unstructured variance–covariance, compound symmetry, and antedependent models. We will therefore present our results using the Toeplitz modeling for the repeated measures (over time) for our ANCOVA main and interaction results. As the TCD measure is known to decrease over time, irrespective of treatment, we used the patient’s age at each episode to be a covariate, controlling for age-related changes in arterial velocities on TCD. The average age by treatment category was 6.92, 8.75, and 9.67 for no treatment, HU, and CT, respectively, and 7.47 overall. Both HU and CT patients would be expected to have lower arterial velocities based on age difference alone. Using mixed methods modelling, we assessed change in MCA TAMX velocities from a single patient over time while controlling for age as a covariate. The analysis was conducted as follows.

The main model (between-subject treatment), covariate, and left–right (LR) effects were assumed to be fixed, while the episode effect and treatment–episode interaction was estimated within a subject. Thus, we have a repeated (in time) measurement ANCOVA-like model. We analyzed the effect of treatment by taking a mixed model framework with repeated, longitudinal measures, and with patient age at TCD as a covariate. We used a general linear mixed model (repeated-measure ANCOVA with LR measures taken at each episode). TCD was modeled as a function of treatment, episode time, their interaction, LR impact, and age at episode covariation. More specifically:

$$y_{ijkt}=\mu \;+\;\alpha_{i}\;+\;\tau_{t}\;+\;{\left(\alpha \tau \right)}_{it}\;+\;\delta_{itk}\;+\;\gamma x_{ijt}\;+\;\epsilon_{ijkt}$$

For the kth measure ***j*** = 1, 2 for LR of the ***j***th patient taking TCDs at time ***t***, and ***α_i_*** is the treatment effect at level: (1) no treatment, (2) CT, and (3) HU for patient ***j***. (***ατ***)***_it_*** represents the interaction effect of the treatment type with episode time. $\delta_{itk}$ represents the effect of LR of treatment ***k*** = 1, 2 over time, episodes, and treatments. $x_{ijt}$ is the age of patient ***j*** of treatment ***i*** at time ***t***. The error term is assumed to be unbiased and independently distributed (over time, after adjusting for patient age) with a constant variance, i.e., $\epsilon_{ijkt}$ ~ *N* (0,*σ*2). More detailed methodology is described in the Supplement file.

## 3. Results

Of the 329 patients included in the study, 111 patients had used HU at the time of at least one TCD, and 218 had never been on HU during a TCD. The approach to HU prescription changed over the course of the study, particularly with the reports of the Baby Hug data [21]. Early on, HU was prescribed for patients with severe disease (multiple pain admissions or acute chest syndrome), although many refused. Later, around 2011 with the reports of the Baby Hug data [21], HU began to be routinely recommended in children over 2 years old, with allowance of use over 6 months of age. During this time, the primary differentiator was patient/caregiver choice. The exact extent of this prescription change could not be fully assessed during the chart review. We recognize this as an unavoidable confounder of our study. Table 1 provides summarized information comparing patients who were receiving HU at the time of at least one TCD and patients who did not receive HU during the course of our study. The majority of patients in our study had never taken HU. Additionally, HU treatment at the time of TCD was associated with older patient age, which is itself associated with lower MCA TAMX velocities. This important factor makes accounting for age as a covariate vital to fully assess the effect of HU treatment on MCA velocity.

**Figure 2:**
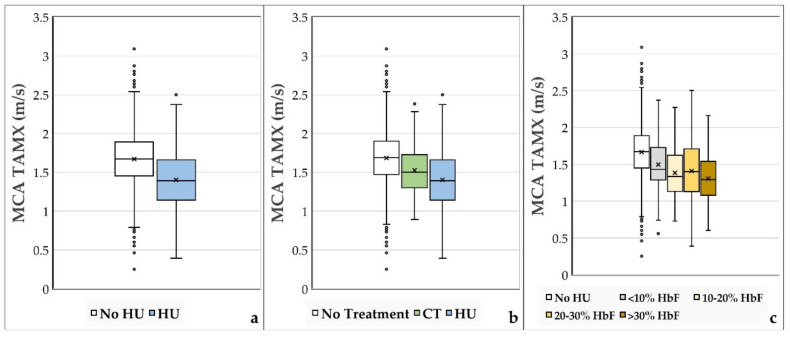
(**a**–**c**): MCA TAMX Values. Figure 2a,b compares the TAMX velocities based on treatment type at the specific time of each TCD. (**a**) No HU vs. HU at the time of TCD; (**b**) no treatment vs. CT vs. HU received at the time of TCD; (**c**) TAMX values compared by Hemoglobin F (% of total hemoglobin that is HbF)—provides an estimate of HU adherence. The primary mechanism of HU in SCD treatment is increasing the proportion of HbF in the blood to prevent sickling. HbF measurements are commonly used for monitoring adherence to HU. Increased HbF indicates good adherence to treatment and was found to be associated with lower TAMX velocities. Plots were constructed using median [midline], mean [‘x’], and interquartile range (IQR) [box], with outliers beyond 1.5*IQR from the nearest quartile.

From the 329 patients in our study, 504 TAMX velocities were obtained during HU treatment, 248 velocities during CT treatment, and 2188 velocities in the absence of either treatment. Figure 2a compares TAMX velocities from patients taking HU with patients not taking HU, regardless of other interventions. The *t*-test demonstrated a statistically significant difference between the two groups, with HU treatment associated with lower MCA TAMX values. Figure 2b compares the TAMX velocities between HU treatment, CT treatment, and no treatment. As depicted in Figure 2b, the mean MCA TAMX velocity in the HU treatment group was 1.40 +/− 0.34 m/s (*n* = 504); the mean MCA TAMX velocity in the CT group was 1.53 +/− 0.29 m/s (*n* = 248); and in the no treatment group, the mean MCA TAMX velocity was 1.68 +/− 0.34 m/s (*n* = 2188). ANOVA testing demonstrated the presence of a significant difference between means, and post hoc *t*-tests comparing each set showed all of the differences between means to be significant (all had *p* < 0.01). Figure 2c shows MCA TAMX velocities as a function of HbF values, a measure of patient compliance and aggressiveness of prescriber dosing. Higher HbF values were associated with lower MCA velocities, supporting the associations shown in Figure 2a,b as being truly related to the HU effect rather than merely coincidental in a studied population that was likely variably compliant with prescribed HU.

Table 2 provides a summary of values obtained from each treatment group in our subsequent mixed model analysis, as well as the mean age for each group at the time of TCD.

**Figure 3:**
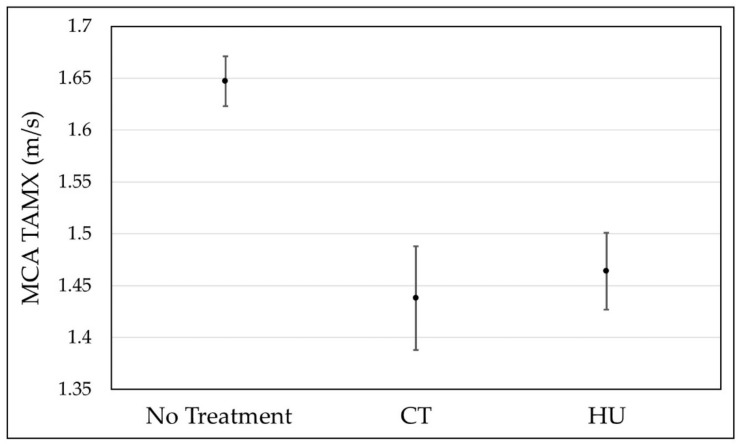
Mean MCA TAMX velocities—controlled for age. Graphic depiction of least squares means estimates for the three treatment groups, accounting for age as a covariate. The black dots indicate Mean MCA TAMX velocities, and the vertical lines indicate 95% confidence intervals. Age at time of TCD was higher for CT (9.67 years) and HU (8.75 years) treatment groups compared with no treatment (6.92). Because increasing age is associated with improved cerebral dynamics (i.e., lower TAMX velocities), performing the analysis with age as a covariate was vital. While accounting for age, the MCA TAMX values as measured using TCDs were significantly lower for both the CT and HU groups.

Table 3 and Figure 3 demonstrate the results of our mixed methods modelling, assessing the MCA TAMX values for individual patients assessed in our study. This analysis treated age as a covariate in order to demonstrate the change in values seen in each treatment group, corrected for change in age. This approach demonstrated that increasing age was associated with lower MCA TAMX values.

While controlling for age as a covariate, the mean TAMX value for HU treatment group was 1.464 m/s, representing an 11.2% decrease when compared with the no treatment group. The 95% confidence intervals (CI) of the no treatment group and HU group do not overlap, demonstrating a statistically significant decrease in TAMX values with HU use, independent of the effect of age. Notably, the CIs of the HU group and transfusion group overlapped, demonstrating no statistical difference between their associations with TCD outcomes.

Based on the displayed TAMX values in Table 4, HU treatment is associated with frequencies of 81.1% normal, 15.7% conditional, and 3.4% abnormal measurements. In comparison, no treatment is associated with 50.2% normal, 34.2% conditional, and 15.5% abnormal measurements. The frequency of abnormal TAMX measurements was 4.56 times greater in patients with no treatment than in those receiving HU therapy. The Chi-square analysis also showed this difference to be statistically significant (*p* < 0.01).

## 4. Discussion

In this retrospective chart review, we tested the hypothesis that HU is associated with a decrease of >10% MCA TAMX velocity compared with children receiving no treatment, when accounting for age as a covariate. HU use in children with SCD at the Children’s Hospital of Michigan in Detroit was found to be associated with MCA TAMX velocities 11.2% lower than the values obtained in the absence of treatment, while accounting for age as a covariate using mixed methods modelling. This association was found to represent a statistically significant difference. Because chronic transfusions are indicated in patients with abnormal TAMX scores (>2 m/s), frequencies of abnormal values by treatment type are clinically important. Our data showed HU treatment to be associated with a 3.4% frequency of abnormal TAMX measurements compared with 15.5% among those receiving no treatment, representing a risk ratio of 0.219. Age was also necessarily accounted for due to the tendency of MCA TAMX velocities to decrease with increasing age, even in the absence of intervention. The least-square mean difference results we describe were derived after adjusting for both the age covariate and the time dependent error modeling of the Toeplitz method, accounting for both age and duration of treatment. The data from our study suggest that primary prophylactic HU treatment in children with HbSS SCD is associated with lower maintenance TCD velocities when compared with no treatment, strengthening the already extensive evidence in favor of the early use of hydroxyurea for children with sickle cell disease, to prevent progression to abnormal MCA TAMX velocities (>2 m/s) and the subsequent need for CT therapy. Figure 3 also demonstrates that TCD velocities were not statistically different between the HU and CT groups when accounting for patient age at the time of TCD.

Because HU treatment is associated with lower cerebral blood flow velocities—a metric specifically used for determining CT indication—we may also state that HU is associated with a decreased need for CT therapy and its associated costs and complications. Furthermore, there are many places in the world where pediatric SCD patients do not have access to CT treatment. HU provides a much cheaper and more accessible alternative that, although not proven to be non-inferior to CT for primary stroke prophylaxis, does show evidence of being associated with lower TAMX velocities, which is likewise associated with a decreased stroke risk [3].

Another consideration in HU primary prophylaxis is dosage. HU dosage in SCD treatment has been recently explored in Abdullahi’s [19] clinical trial conducted in Nigeria, where they demonstrated that low-dose (10 mg/kg/day) and moderate-dose (20 mg/kg/day) HU treatment resulted in comparable stroke risk, but the moderate-dose group had decreased all-cause hospitalizations. Our study did not assess the effects of HU dosage on MCA TAMX scores. Instead, we titrated HU to the maximum tolerated dose for each patient according to established treatment guidelines. Of note, the mean HU dose in our study was 24.7+/− 5.3 mg/kg/day, with 80.5% of patients taking a dose of at least 20 mg/kg/day. We would suggest that the results of our HU treatment group would be more comparable to the moderate-dose HU treatment group than the low-dose group in Abdullahi’s study, with the understanding that their study was a randomized controlled trial and ours is a retrospective chart review.

Additionally, measures of HU adherence/effect (Figure 2c) provided evidence to support the logical assumption that successfully targeting higher HbF levels via more aggressive dosing and increased compliance with daily HU regimen is associated with decreased TCD velocities. Daily compliance with HU should be addressed by practitioners, and barriers to adherence should be regularly investigated, as our study suggests an association between HU compliance and lower MCA blood flow velocities, which is associated with a decreased stroke risk [3].

While our analysis resulted in statistically significant findings suggesting an advantage of prophylactic HU treatment in children with SCD, it has limitations. It is a retrospective study and thus lacks randomization of the treatment groups. Given the results of previous research trials demonstrating the efficacy of CT and HU therapy in secondary stroke prophylaxis for children with SCD [18], a randomized controlled trial addressing our research question would have ethical challenges. Because of its retrospective approach, this study can only imply association and not causation. Additionally, we did not directly assess stroke incidence as our measured outcome, but rather MCA blood flow velocity. This was done for two reasons. First, MCA blood flow velocity has been shown to be a reliable surrogate for stroke risk in children with SCD [3]. Second, stroke as a direct outcome would be subject to bias as children in the United States who are found to have elevated MCA velocities are promptly treated with transfusion therapy, mitigating stroke incidence regardless of prior treatment. However, because our study does not measure stroke incidence directly, we can only claim to demonstrate an association between prophylactic HU and decreased MCA blood flow velocities, rather than decreased stroke risk. We are also, therefore, unable to conclude that HU is non-inferior to CT therapy in stroke prevention among children with SCD. Finally, our study was subject to inherent selective bias regarding HU treatment. During the latter part of our study’s time frame, HU was offered to all patients who fit the inclusion criteria. Early on, however, HU was primarily prescribed to patients with severe disease (acute chest syndrome or multiple admissions due to pain). Patients with more severe phenotypes would be expected to have more significant vasculopathy and higher TCD values compared with those receiving no treatment. This selective bias would be expected to influence the HU treatment group in our study to have higher TAMX values. The fact that they were still significantly lower than the values in the group without treatment would grant further support to our stated conclusions.

## 5. Conclusions

The results of our retrospective chart review support the findings of previous studies, demonstrating that HU use in children with SCD is associated with decreased MCA TAMX velocities, corrected for age, as measured by TCD. Given the previously established association between TCD results and stroke risk [3], it may be inferred that our study provides further evidence that HU is associated with a decreased risk of stroke in the pediatric SCD population. Additionally, our study showed that the frequency of abnormal TAMX values (>2 m/s) was 4.56 times greater in those receiving no treatment compared with those receiving HU. Because abnormal measurements are an indication for CT therapy, this comparison is directly relevant to clinical practice. The evidence is already strong for HU as primary prophylaxis following abnormal TCD and stabilization of vasculopathy via a period of recurrent transfusion therapy, but the evidence for its role in preventing stroke outside of this scenario has continued to be less clear. While this question would be more directly answered with a randomized, controlled trial, obvious ethical concerns make such a study impractical. The results of our retrospective study provide greater clarity that the initiation of prophylactic HU in children with SCD is associated with lower MCA TAMX velocities and a potentially decreased need for chronic transfusion therapy. Given the previously established association between MCA TAMX velocities and stroke risk [3], it can also be inferred that primary HU prophylaxis may be associated with decreased stroke risk among children with SCD, providing further support for the early initiation of HU treatment in this population.

## Figures and Tables

**Table 1:** Summary of patient information. As a retrospective chart review, age was determined to be relevant only in relation to time of treatment and TCD measurements. HU dosage was prescribed based on recommendations at the time and was titrated to a maximum tolerated dose as a function of body weight (kg). History of prior CT therapy was notably higher among those who were never treated with HU at the time of TCD.

	Hydroxyurea (*n* = 111; 34%)	No Hydroxyurea (*n* = 218; 66%)
Sex		
Female	54 (49%)	119 (55%)
Male	57 (51%)	99 (45%)
Age at start of HU, years	7.16 +/− 4.11	N/A
Duration of HU Treatment at time of TCD, years	2.53 +/− 2.15	N/A
Age at time of TCD, years	8.75 +/− 3.65	7.16 +/− 3.61
HU Dosage (mg/kg/day)	24.7 +/− 5.3	N/A
MCA TAMX (m/s)	1.40 +/− 0.34	1.67 +/− 0.33
History of prior CT therapy	20 (18%)	92 (42%)
Abnormal TAMX (>2 m/s) during study, requiring transfusion	5 (4%)	34 (16%)

**Table 2:** Summary of results obtained from the dataset. Table 2 summarizes some of the sample characteristics by treatment level: no treatment, CT, and HU. The 329 unique patients were distributed by no treatment (167), CT (70), and HU (92), with a maximum of ten episodes per patient, or 1094, 124, and 252 episodes, respectively, and totaling 1470 episodes. The average number of episodes per patient is depicted by treatment category and overall average. Each patient’s TCD was measured twice at each episode, doubling the number of TCD measures. We assume that each side (denoted LR) TCD represents hemispherical stroke risk, not a repeated measure of general stroke risk.

	No Treatment	CT	Hydroxyurea	Total
#Patients	167	70	92	329
Number of TCDs	1094	124	252	1470
Mean Number of TCDs/Patient	6.55	1.77	2.74	4.47
Mean Age at TCD	6.92	9.67	8.75	7.47
Mean MCA TAMX	1.69	1.54	1.37	1.62

**Table 3:** Mean MCA TAMX velocities—controlled for age. Least squares means estimates for the three treatment groups, factoring in age effects as a covariate for analyzing the TCD results. The 95% confidence intervals between no treatment and HU do not overlap, demonstrating a statistically significant difference between treatment groups. The results from this table are graphically depicted in Figure 3, below.

	Mean TAMX	SE	DF	Lower 95%	Upper 95%
No Treatment	1.648	0.012	689	1.624	1.672
CT	1.437	0.025	2590	1.388	1.487
HU	1.464	0.019	1681	1.427	1.501

**Table 4:** Contingency table of TAMX categories. This contingency table displays total number of TAMX measurements categorized by treatment type at the time of TCD, allowing for a comparison of the frequency of normal, conditional, and abnormal TAMX velocities. HU treatment was associated with more “normal” TAMX scores and less “abnormal” TAMX scores when compared with no treatment.

Treatment	Normal (<1.7 m/s) MCA TAMX	Conditional (1.7–2.0 m/s) MCA TAMX	Abnormal (>2.0 m/s) MCA TAMX	Total
HU	408	79	17	504
CT	172	54	22	248
No Treatment	1099	749	340	2188
Total	1679	882	379	2940

## Data Availability

The data presented in this study are available in the Supplementary Materials.

## Supplementary Materials

The following supporting information can be downloaded at: https://www.mdpi.com/article/10.3390/jcm11123491/s1, Table S1: Study Summary Results; Table S2: Model Fitting Results for Repeated-Measure Mixed Models; Figure S1: Mean MCA TMAX Velocities, Controlled for age, Least Squares Means Plot.

## References

[1] CDC Data & Statistics on Sickle Cell Disease|CDC. https://www.cdc.gov/ncbddd/sicklecell/data.html.

[2] Jordan L.C., Casella J.F., Debaun M.R. (2012). Prospects for primary stroke prevention in children with sickle cell anaemia. Br. J. Haematol..

[3] Adams R.J., McKie V.C., Carl E.M., Nichols F.T., Perry R., Brock K., McKie K., Figueroa R., Litaker M., Weiner S. (1997). Long-term stroke risk in children with sickle cell disease screened with transcranial Doppler. Ann. Neurol..

[4] Adams R.J., Brambilla D.J., Granger S., Vichinsky E., Abboud M.R., Pegelow C.H., Woods G., Rohde E.M., Nichols F.T., Jones A. (2004). Stroke and conversion to high risk in children screened with transcranial Doppler ultrasound during the STOP study. Blood.

[5] Sarnaik S. (1979). Periodic transfusions for sickle cell anemia and CNS Infarction. Arch. Pediatr. Adolesc. Med..

[6] Russell M.O., Goldberg H.I., Reis L., Friedman S., Slater R., Reivich M., Schwartz E. (1976). Transfusion therapy for cerebrovascular abnormalities in sickle cell disease. J. Pediatr..

[7] Adams R.J., Mckie V.C., Brambilla D., Carl E., Gallagher D., Nichols F.T., Roach S., Abboud M., Berman B., Driscoll C. (1998). Stroke Prevention Trial in Sickle Cell Anemia. Control. Clin. Trials.

[8] National Heart, Lung, and Blood Institute, National Library of Medicine (U.S.) (1997). Clinical Alert: Periodic Transfusions Lower Stroke Risk in Children with Sickle Cell Anemia. https://www.nlm.nih.gov/databases/alerts/sickle97.html.

[9] Ohene-Frempong K., Weiner S.J., Sleeper L.A., Miller S.T., Embury S., Moohr J.M., Wethers D.L., Pegelow C.H., Gill F.M., The Cooperative Study of Sickle Cell Disease (1998). Cerebrovascular accidents in sickle cell disease: Rates and risk factors. Blood.

[10] Bernaudin F., Verlhac S., Arnaud C., Kamdem A., Chevret S., Hau I., Coïc L., Leveillé E., Lemarchand E., Lesprit E. (2011). Impact of early transcranial Doppler screening and intensive therapy on cerebral vasculopathy outcome in a newborn sickle cell anemia cohort. Blood.

[11] Adams R.J., Brambilla D. (2005). Optimizing Primary Stroke Prevention in Sickle Cell Anemia (STOP 2) Trial Investigators. Discontinuing prophylactic transfusions used to prevent stroke in sickle cell disease. N. Engl. J. Med..

[12] Agrawal R.K., Patel R.K., Shah V., Nainiwal L., Trivedi B. (2013). Hydroxyurea in Sickle Cell Disease: Drug Review. Indian J. Hematol. Blood Transfus..

[13] Gulbis B., Haberman D., Dufour D., Christophe C., Vermylen C., Kagambega F., Corazza F., Devalck C., Dresse M.-F., Hunninck K. (2005). Hydroxyurea for sickle cell disease in children and for prevention of cerebrovascular events: The Belgian experience. Blood.

[14] Kratovil T., Bulas D., Driscoll M.C., Speller-Brown B., McCarter R., Minniti C.P. (2006). Hydroxyurea therapy lowers TCD velocities in children with sickle cell disease. Pediatr. Blood Cancer.

[15] Zimmerman S.A., Schultz W.H., Burgett S., Mortier N.A., Ware R.E. (2007). Hydroxyurea therapy lowers transcranial Doppler flow velocities in children with sickle cell anemia. Blood.

[16] Lagunju I., Brown B.J., Oyinlade A.O., Asinobi A., Ibeh J., Esione A., Sodeinde O.O. (2019). Annual stroke incidence in Nigerian children with sickle cell disease and elevated TCD velocities treated with hydroxyurea. Pediatr. Blood Cancer.

[17] Rankine-Mullings A., Reid M.E., Soares D., Taylor-Bryan C., Wisdom-Phipps M., Aldred K., Latham T., Knight-Madden J., King L., Badaloo A. (2019). Hydroxyurea therapy to prevent incident stroke among children with sickle cell anaemia in Jamaica: The extend trial. Blood.

[18] Ware R.E., Davis B.R., Schultz W.H., Brown R.C., Aygun B., Sarnaik S., Odame I., Fuh B., George A., Owen W. (2016). Hydroxycarbamide versus chronic transfusion for maintenance of transcranial doppler flow velocities in children with sickle cell anaemia-TCD with Transfusions Changing to Hydroxyurea (TWiTCH): A multicentre, open-label, phase 3, non-inferiority trial. Lancet.

[19] Abdullahi S.U., Jibir B.W., Bello-Manga H., Gambo S., Inuwa H., Tijjani A.G., Idris N., Galadanci A., Hikima M.S., Galadanci N. (2022). Hydroxyurea for primary stroke prevention in children with sickle cell anaemia in Nigeria (SPRING): A double-blind, multicentre, randomised, phase 3 trial. Lancet Haematol..

[20] Weisman J.K., Diamond C.E., Kappa S., Nickel R.S. (2020). Transcranial Doppler Screening Adherence among Children with Sickle Cell Anemia Seen in the Emergency Department. J. Pediatr..

[21] Wang W.C., Ware R.E., Miller S.T., Iyer R.V., Casella J.F., Minniti C.P., Rana S., Thornburg C.D., Rogers Z.R., Kalpatthi R.V. (2011). Hydroxycarbamide in very young children with sickle-cell anaemia: A multicentre, randomized, controlled trial (BABY HUG). Lancet.

