# Supplementary material for: Prophylactic Hydroxyurea Treatment Is Associated with Improved Cerebral Hemodynamics as a Surrogate Marker of Stroke Risk in Sickle Cell Disease: A Retrospective Comparative Analysis
Source: J Clin Med. 2022 Jun 17;11(12):3491. doi: 10.3390/jcm11123491 (PMC9225168; doi:10.3390/jcm11123491)
Supplement: Supplementary file 1 [file jcm-11-03491-s001.zip › jcm-1761335-supplementary.pdf]

Study Methods & Results:

Table S1. Study Summary Results.

Variable	No Treatment	CT Treatment	HU Treatment	Total
Patients	167	70	92	329
Episodes	1,094	124	252	1,470
Measures	2,188	248	504	2,940
Episodes/ Patient	6.55	1.77	2.74	4.47
Average age at Treatment	6.92	9.67	8.75	7.47
Average Measurement	1.69	1.54	1.37	1.62

Table S1 summarizes some of the sample characteristics by treatment level: No Treatment, CT, and HU. The 329 unique patients were distributed by No Treatment (167), CT (70), and HU (92), with a maximum of ten episodes per patient, or 1,094, 124 and 252 episodes, respectively, and totaling 1,470 episodes. The average number of episodes per patient is depicted by treatment category and overall average. Each patient's TCD was measured twice at each episode, doubling the number of TCD measures. We assume that each side (denoted LR) TCD represents hemispherical stroke risk, not a repeated measure of general stroke risk.

The number of episodes varied by patient and was set to a maximum of ten episodes to assure that each treatment level was represented at each episode (i.e., at event time t). The repeated time measures (within subject) are assumed to be correlated. Several reasonable variance-correlation structures are first estimated, the best fitting one selected before the main model effects are derived. The most general form of variance-covariance modeling (Unstructured model), requiring here a 10 episodic variances and n(n-1), or in our case 45 unique covariances. For our data, the following matrix represents this structure:

Variance-Covariance Matrix (SE) of Unstructured System for Repeated Time Measures

Covariance Matrix (SE)

Row	01	02	03	04	05	06	07	08	09	10
01	0.1189 (0.0069)									
02	0.0833 (0.0062)	0.1276 (0.0078)								
03	0.0662 (0.0059)	0.0786 (0.0064)	0.1066 (0.0073)							
04	0.0581 (0.006)	0.063 (0.0064)	0.0688 (0.0063)	0.0966 (0.0074)						
05	0.0522 (0.0066)	0.0523 (0.0069)	0.0594 (0.0068)	0.0556 (0.0064)	0.0860 (0.0079)					
06	0.0526 (0.0081)	0.0534 (0.0084)	0.0517 (0.0081)	0.0518 (0.0075)	0.0512 (0.0072)	0.0928 (0.01)				
07	0.054 (0.009)	0.0568 (0.0096)	0.0534 (0.0096)	0.0534 (0.0087)	0.0593 (0.0086)	0.0661 (0.0092)	0.1015 (0.0121)			
08	0.0533 (0.0111)	0.054 (0.0125)	0.0487 (0.0127)	0.0415 (0.0118)	0.0353 (0.0103)	0.048 (0.0107)	0.0608 (0.0115)	0.0956 (0.0154)		
09	0.074 (0.0143)	0.0792 (0.0159)	0.0672 (0.017)	0.0589 (0.0156)	0.0426 (0.0149)	0.0527 (0.0142)	0.0568 (0.0139)	0.0719 (0.0157)	0.1262 (0.0241)	
10	0.1022 (0.0219)	0.1076 (0.024)	0.0906 (0.0262)	0.071 (0.022)	0.0956 (0.0226)	0.0954 (0.022)	0.0876 (0.0218)	0.0872 (0.0222)	0.1004 (0.0281)	0.2203 (0.0584)

Since the lowest variance estimate (0.0860) times four (heuristically, about 2 standard derivations) is 0.3440, which is greater than the maximum variance estimate of 0.2203, we assume a constant variance model for the time repeated measures. At the other end of the spectrum is the compound symmetry model, which requires the estimate of only two parameters: a variance and a single covariance representing an equal correlation (or covariation) for all lags to t. The models representing intermediate positions are the Antedependent, Toeplitz and AR(1) models. All of the models were estimated under their constant variance versions. The off-diagonal correlations show no discernable or obvious pattern, though not likely a spherically constant given the mixed signs and various magnitudes. We will, therefore, test the four constant variance structures available in JMP Mixed Models for repeated time measures and select the best fitting result. These are:

The following Table YY reflects JMP mixed model goodness-of-fit measures for REML estimation of the constant variance models:

Table S2.: Model Fitting Results for Repeated-Measure Mixed Models.

Model	-2 Residual Log Likelihood	-2 Log Likelihood	AICc	BIC
Unstructured	362.547	294.741	423.544	797.869
Compound Symmetry	516.742	448.375	468.450	528.237
Antedependent	555.467	488.906	525.140	632.657
**Toeplitz**	427.324	360.022	396.256	503.773
AR(1)	565.895	499.334	519.409	579.195

The AICc and BIC columns represent fit measures that penalize the use of more parameter estimates, so that even though the log likelihood measures are best (lowest) for the Unstructured model, the Toeplitz model has the best fit measures overall.

The Toeplitz modeling, which estimates a constant covariance (correlation) over a given lag, but no pattern over different lags is best fitting. Although the least-restrictive (but one with the most parameters to estimate), the Unstructured variance-covariance model provides some AICc fit, Toeplitz is better over the four fit statistic measures. The other variance-covariance models perform relatively poorly. We will therefore present our results using the Toeplitz modeling for the repeated measures (over time) for our repeated measure ANCOVA main and interaction results.

Since the TCD measure is known to decrease over time, irrespective of treatment, we used the patient's age at each episode to be a covariate, whose estimated parameter controls for this non-treatment impact on TCD. Table S1 shows the average age by treatment category as 6.92, 8.75, and 9.67 for No treatment, HU and CT, respectively, and 7.47 overall. Both HU and CT patients would be expected to be lower vis-à-vis the no treatment group base on their age at treatment alone. TCD mean differences across the treatment groups indicate that CT is significantly lower (better) than the no treatment group mean, while significantly higher than HU treatment.

The Model:

The main model (between-subject treatment), covariate and LR effects are assumed to be fixed, while the episode effect and treatment-episode interaction is

estimated within subject. Thus, we have a repeated (in time) measurement ANCOVA-like model.

We analyze the effect of treatment by taking a mixed model framework with repeated, longitudinal measures, and with patient age at episode as a covariate. We use a general linear mixed model (repeated-measure ANCOVA with LR measures taken at each episode). TCD is modeled as a function of treatment, episode time, their interaction, LR impact and age at episode covariation. More specifically:

$$y_{ijkt} = \mu + \alpha_i + \tau_t + (\alpha\tau)_{it} + \delta_{itk} + \gamma x_{ijt} + \epsilon_{ijkt}$$

for the kth measure j=1, 2 for LR of the jth patient taking TCDs at time t. α_i is the treatment effect at level: 1) No treatment 2) CT, and 3) HU for patient j. $(\alpha\tau)_{it}$ represents the interaction effect of treatment type with episode time. δ_{itk}represents the effect of LR of treatment k=1, 2 over time episodes and treatments. x_{ijt} is the age of patient j of treatment i at time t. The error term is assumed to be unbiased and independently distributed (over time, after adjustment and patients) with a constant variance, i.e. $\varepsilon_{ijkt} \sim N(0,\sigma^2)$.

<u>Model Results:</u>

The Fixed effects results are:

Toeplitz Model Parameter Estimates:

Term	Estimate	Std Error	DFDen	t Ratio	Prob>|t|	95% Lower	95% Upper
Intercept	1.6944367	0.0258755	1803.1	65.48	<0.0001*	1.6436876	1.7451858
Age_Trt	-0.021079	0.0025679	1216.0	-8.21	<0.0001*	-0.026117	-0.016041
Category[No Treatment]	0.13115	0.0106066	2669.0	12.36	<0.0001*	0.110352	0.1519481
Category[CT]	-0.078918	0.0167538	2547.9	-4.71	<0.0001*	-0.111771	-0.046066
E_num	-0.005671	0.0043231	852.8	-1.31	0.1899	-0.014156	0.0028138
(E_num-3.63197)*Category[No Treatment]	0.0188382	0.0037312	1415.6	5.05	<0.0001*	0.011519	0.0261574
(E_num-3.63197)*Category[CT]	-0.012974	0.0060161	1464.5	-2.16	0.0312*	-0.024775	-0.001173
RorL[L]	0.0139051	0.0112407	549.3	1.24	0.2166	-0.008175	0.0359851

All terms are significant at traditional levels except for the time period and LR effects, though time period and treatment categories significantly interact. As suspected the parameter estimate for age at treatment is negative. This characterization is confirmed by the following more summarized table of fixed effects.

Fixed Effects Tests

Source	Nparm	DFNum	DFDen	F Ratio	Prob > F
Age_Trt	1	1	1235.2	66.449001	<0.0001*
Category	2	2	2603.1	79.317771	<0.0001*
E_num	1	1	859.9	1.9386715	0.1642
E_num*Category	2	2	1121.6	13.137271	<0.0001*
RorL	1	1	561.0	1.6752726	0.1961

The least squares Means estimates of treatment levels is different from the average treatment effects presented in Table S1, where HU (1.37) was significantly lower than CT (1.54), and both were significantly lower than the No Treatment group average of 1.69.

Multiple Comparisons for Category

Least Squares Means Estimates

Category	Estimate	Std Error	DF	Lower 95%	Upper 95%
No Treatment	1.6475659	0.01223064	688.64	1.6235520	1.6715797
CT	1.4374975	0.02542980	2590.3	1.3876327	1.4873623
HU	1.4641842	0.01871844	1681.4	1.4274703	1.5008981

The plot of these least square mean differences is depicted as follows:

Mean MCA TMAX Velocities, Controlled for age

Least Squares Means Plot

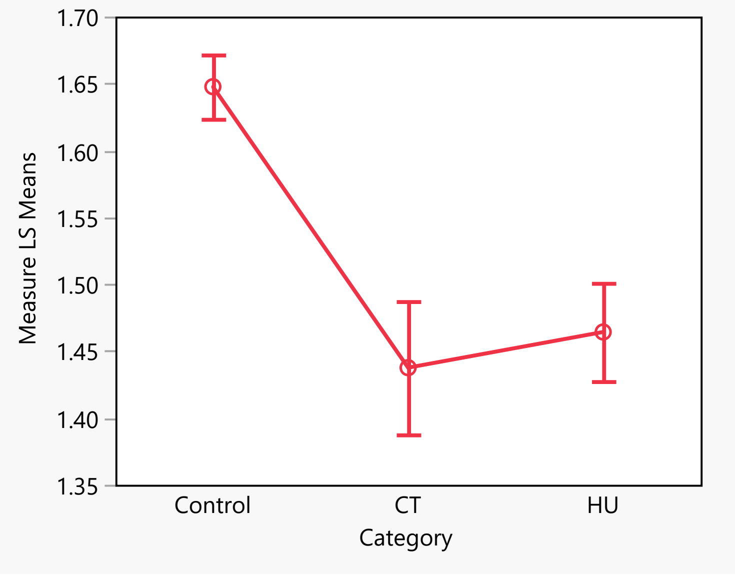

Figure S1.: Graphic depiction of least squares means estimates for the three treatment groups, accounting for age as a covariate. MCA TMAX values as measured by TCDs.

The mean difference plots indicate that both CT and HU treatments are better than the no treatment group. CT appear to have insignificantly lower (better) results, but also has higher dispersion of results. The following post-hoc Tukey pairwise mean differences similarly indicate that, although both treatment modes are better than No Treatment, they are not significantly different from one another. Given this indifference, cost and patient invasiveness considerations should dictate the clinical choice.

Tukey HSD All Pairwise Comparisons

Quantile = 2.34506, Adjusted DF = 2573.9, Adjustment = Tukey-Kramer

All Pairwise Differences

Category	-Category	Difference	Std Error	t Ratio	Prob>|t|	Lower 95%	Upper 95%
No Treatment	CT	0.210068	0.0242782	8.65	<0.0001*	0.153135	0.2670022
No Treatment	HU	0.183382	0.0183906	9.97	<0.0001*	0.140255	0.2265086
CT	HU	-0.026687	0.0290307	-0.92	0.6281	-0.094765	0.0413921

Filename: jcm-1761335-supplementary.docx
Directory: E:\6.17\remotesensing-1722081
Template:
C:\Users\MDPI\AppData\Roaming\Microsoft\Templates\Normal.dotm
Title:
Subject:
Author: Joe Callaghan
Keywords:
Comments:
Creation Date: 6/16/2022 11:25:00 PM
Change Number: 3
Last Saved On: 6/17/2022 1:43:00 PM
Last Saved By: MDPI
Total Editing Time: 12 Minutes
Last Printed On: 6/17/2022 5:32:00 PM
As of Last Complete Printing
Number of Pages: 6
Number of Words: 1,516 (approx.)
Number of Characters: 8,627 (approx.)
